# The role of algorithms in guiding emergency airway management

**DOI:** 10.1002/anr3.12117

**Published:** 2021-05-07

**Authors:** B. A. McGrath, T. E. Heaton

**Affiliations:** ^1^ Department of Anaesthesia and Critical Care Manchester University NHS Foundation Trust Manchester UK

Stangoe et al. describe the successful management of a rare cause of tracheostomy tube ‘ball‐valve’ obstruction [1]. The authors followed the widely used emergency management algorithms developed by the National Tracheostomy Safety Project (NTSP) [2] and have correctly highlighted that such algorithms may not account for every possible scenario.

The NTSP took a transparent approach to guideline development, detailing the methodologies [2], testing and evaluation and measuring the impact of implementation [3]. The algorithms were designed to address the commonest scenarios identified from forensic analysis of reported incidents [4]. The Working Parties agreed that the benefits of a single generic algorithm focused on common and easily reversible emergency situations was preferable to multiple algorithms addressing problem‐specific, patient‐specific or location‐specific approaches. Adopting a generic approach simplifies and standardises teaching and training while addressing the vast majority of the causes of tracheostomy emergencies. Accepting that special circumstance would inevitably be encountered, adherence to critical airway management principles is still likely to benefit the patient, as in the case described. Even if a suction catheter can be passed, the responder is guided to “*consider partial airway obstruction*” while continuing to assess the airway. Appropriate next steps when faced with an inability to ventilate a patient who has a tracheostomy in situ are cuff deflation; endoscopic evaluation if the clinical situation allows; removal of the tracheostomy tube; all followed by re‐assessment and increasingly invasive attempts to oxygenate by either airway.

We commend Dr Stangoe and colleagues for their effective management of this highly challenging airway emergency. We encourage readers to always consider any emergency algorithm in conjunction with the supporting information, full manuscript and educational resources.
